# Does admission prevalence change after reconfiguration of inpatient services? An interrupted time series analysis of the impact of reconfiguration in five centres

**DOI:** 10.1186/s12913-021-06070-7

**Published:** 2021-01-21

**Authors:** Joanne Martin, Edwin Amalraj Raja, Steve Turner

**Affiliations:** 1 Child Health, University of Aberdeen, Aberdeen, AB25 2ZG Scotland; 2 Medical Statistics Team, University of Aberdeen, Aberdeen, Scotland

**Keywords:** Admission, Child, Hospital, Reconfiguration

## Abstract

**Background:**

Service reconfiguration of inpatient services in a hospital includes complete and partial closure of all emergency inpatient facilities. The “natural experiment” of service reconfiguration may give insight into drivers for emergency admissions to hospital. This study addressed the question does the prevalence of emergency admission to hospital for children change after reconfiguration of inpatient services?

**Methods:**

There were five service reconfigurations in Scottish hospitals between 2004 and 2018 where emergency admissions to one “reconfigured” hospital were halted (permanently or temporarily) and directed to a second “adjacent” hospital. The number of emergency admissions (standardised to /1000 children in the regional population) per month to the “reconfigured” and “adjacent” hospitals was obtained for five years prior to reconfiguration and up to five years afterwards. An interrupted time series analysis considered the association between reconfiguration and admissions across pairs comprised of “reconfigured” and “adjacent” hospitals, with adjustment for seasonality and an overall rising trend in admissions.

**Results:**

Of the five episodes of reconfiguration, two were immediate closure, two involved closure only to overnight admissions and one with overnight closure for a period and then closure. In “reconfigured” hospitals there was an average fall of 117 admissions/month [95% CI 78, 156] in the year after reconfiguration compared to the year before, and in “adjacent” hospitals admissions rose by 82/month [32, 131]. Across paired reconfigured and adjacent hospitals, in the months post reconfiguration, the overall number of admissions to one hospital pair slowed, in another pair admissions accelerated, and admission prevalence was unchanged in three pairs. After reconfiguration in one hospital, there was a rise in admissions to a third hospital which was closer than the named “adjacent” hospital.

**Conclusions:**

There are diverse outcomes for the number of emergency admissions post reconfiguration of inpatient facilities. Factors including resources placed in the community after local reconfiguration, distance to the “adjacent” hospital and local deprivation may be important drivers for admission pathways after reconfiguration. Policy makers considering reconfiguration might consider a number of factors which may be important determinants of admissions post reconfiguration.

## Background

Emergency paediatric admissions are rising in the UK [1, 2] and research is required into this phenomenon. Partial or complete closure of inpatient facilities (i.e. reconfiguration) has an obvious impact on emergency admissions in the reconfigured and adjacent facilities. There are several reasons why medical facilities may reconfigure including cost, workforce and technology [3–5]. The study of emergency admissions before and after this change may give insight into referral pathways and also inform planning for future closures. Reconfiguration of inpatient services means that some or all unscheduled paediatric care moves to an adjacent hospital and what is not known is whether admission prevalence changes after reconfiguration.

When a ward closes there is an obvious reduction in admissions to that hospital, but how does reconfiguration in on hospital impact on admissions in an adjacent hospital? It is possible that after reconfiguration of services there is more “watchful waiting” by parents and clinicians in the community, and this results in a reduction in admissions to the hospital where inpatient services are now located. Alternatively, reconfiguration of services in one hospital may lower thresholds in the local community for seeking advice and change referral pathways leading to unexpected increase in admissions to adjacent hospitals, and there is evidence of this happening [3]. This latter scenario is problematic since an unexpected increase in the number of admissions to a hospital adjacent to a reconfigured hospital may lead to reduced access to specialists, delays in inpatient care and emergency department overcrowding [6].

A number of paediatric medical wards in Scottish hospitals have been reconfigured since 2004, and either closed to all emergency admissions or have only accepted admissions during the day and been closed overnight. Routinely acquired data can be useful in evaluating the impact of reconfiguration of health services [7]. The aims of this study were (i) to describe changes in emergency admissions to a reconfigured and adjacent hospital post-reconfiguration and (ii) to determine whether there is a change in sum of emergency admissions per month across two hospitals after reconfiguration of inpatient facilities in one of those hospitals. The hypothesis was that the reconfiguration of a paediatric inpatient facility would be followed by an increase in admissions to the reconfigured and adjacent hospitals when combined.

## Methods

### Study overview

Hospitals in Scotland where paediatric inpatient services were reconfigured between 2000 and 2018 were identified. The prevalence of emergency medical admissions/month for hospitals where reconfiguration took place and the adjacent hospital was obtained for the period before and after reconfiguration. The admission rate was expressed as number /1000 children in the regional population per month. The “reconfigured hospital” was defined as the hospital where reconfiguration took place. The “adjacent hospital” was defined as the closest hospital to the reconfigured hospital within the same health board (i.e. geographical region where healthcare is administered by the same authority). Two indices of deprivation were used to categorise inpatient units: first, an index of deprivation (the Scottish Index of Multiple Deprivations) was derived using the postcode for each hospital; second the % of data zones in the local (council) areas which were in the lowest SIMD category( https://www.scotpho.org.uk/life-circumstances/deprivation/data/). Councils were associated with inpatient units as follows: Inverclyde council for Inverclyde Royal Hospital; Perth and Kinross for Perth Royal Infirmary; Moray for Dr Gray’s Hospital; Renfrewshire for Royal Alexandra Hospital; West Lothian for St John’s Hospital; Dundee City for Ninewells Hospital; Aberdeen City for Royal Aberdeen Children’s Hospital; Highland for Inverness; Glasgow City for Royal Hospital for Children (Glasgow); City of Edinburgh for Royal Hospital for Children (Edinburgh). The distance between reconfigured and adjacent hospitals was determined using the AA route planner (https://www.theaa.com/route-planner/route). The separate prevalence of admissions to the reconfigured and the adjacent hospital was determined before and after reconfiguration. The prevalence of combined admissions to reconfigured and adjacent hospitals before and after reconfiguration was determined.

### Admission data

One author (ST) identified hospitals where inpatient services were reconfigured. The lead clinicians in the health board where reconfiguration took place were contacted to determine details of the reconfiguration including the month and year of reconfiguration and the nature of the reconfiguration, e.g. why reconfiguration took place and what contingencies were put in place in the community where reconfiguration took place. Health boards where reconfiguration took place were asked to provide the number of emergency admissions per month of < 16 year olds to all inpatient facilities in that health board for 5 years prior to reconfiguration in one facility and for five years afterwards (or where reconfiguration took place within the last five years, to the most recent data that data were available). An emergency admission in this study was defined as any admission to hospital that was unscheduled and excludes planned paediatric admissions or cases seen and sent home from the emergency department. Data were provided as aggregate numbers and no data cells contained less than five individuals. No individual patient details were available. Ethical approval was not required. The individual health boards whose data were used approved this analysis.

### Inpatient facilities

Reconfiguration took place in five hospitals: Inverclyde Royal Hospital (Greenock), Perth Royal Infirmary (Perth), Royal Alexandra Hospital (Paisley), Dr Gray’s Hospital (Elgin) and St John’s Hospital (Livingston). Figure one displays the location of the reconfigured and adjacent hospitals. Elgin is in the same health board as Aberdeen, and Aberdeen is the designated “adjacent” inpatient facility for Elgin admissions but Elgin is much closer to Inverness than Aberdeen. In recognition that parents from Elgin may present sick children directly to Inverness to avoid travelling to Aberdeen, both Inverness and Aberdeen were considered as “adjacent” hospitals for Elgin. Table 1 gives details of the reconfigured and adjacent hospitals.

**Table 1:** Characteristics of paediatric facilities included in this study. Vigintile scores represent deprivation with 1 being the most deprived and 20 the least deprived

	Details of the reconfigured hospital					Details of adjacent hospital			Distance between reconfigured and adjacent hospitals (miles)
Name (location)	Nature of and date of reconfiguration	Deprivation		Average monthly admission in five years pre-reconfiguration/1000	Name (location)	Deprivation (1=lowest)		Average monthly admission in five years pre-reconfiguration/1000	
		SIMD vigintile (1=lowest)	Density of poverty^a^			SIMD vigintile (1=lowest)	Density of poverty^a^		
Inverclyde Royal Hospital (Greenock)	All inpatient services ceased in Nov ‘04	2	36.8%	3.25	Royal Alexandra Hospital (Paisley)	12	25.3%	4.46	19.3
Perth Royal Infirmary (Perth)	Short stay admission unit operational from Apr ’05 8am-8pm until March ’18 when all inpatient services ceased	20	3.2%	3.78	Ninewells Hospital (Dundee)	18	26.6%	8.47	19.6
Dr Gray’s Hospital (Elgin)	Short stay admission unit operational from Mar ’18 8am-10pm	18	0%	5.70	Royal Children’s Hospital (Aberdeen)	16	6.0%	5.82	65.3
					Raigmore Infirmary (Inverness)	8	5.1%	7.26	37.4
Royal Alexandra Hospital (Paisley)	All inpatient services ceased Dec’17	12	25.3%	5.38	Royal Hospital for Children (Glasgow)	6	44.2%	10.74	6.2
St John’s Hospital (Livingston)	A short stay assessment unit was open from July ’17.	12	13.8%	3.09	Royal Hospital for Children (Edinburgh)	19	8.4%	4.48	16.5

^a^Percentage of “datazones” in the catchment area for the hospital which are in 15% most deprived areas of the SIMD 2016 categorisation

#### Greenock (Inverclyde Royal Hospital, NHS Greater Glasgow and Clyde). 

All inpatient facilities ceased after reconfiguration in Nov 2004 and inpatient facilities were provided in Paisley. No resources were put into the community in Greenock. Data between Dec 1999 and Nov 2009 were analysed.

#### Perth (Perth Royal Infirmary, NHS Tayside)

Round-the-clock inpatient facilities ceased in April 2005 and a short stay admissions unit staffed by paediatricians remained open from 8am-8 pm until March 2018 when all inpatient facilities ceased. The following provisions were put in place for patients in Perth: there were all day clinics at with spaces for rapid reviews (e.g. patients seen the day before); a list faxed to the inpatient facility (Ninewells hospital, Dundee) every evening listing children in the community in Perth who have been in contact with PRI during the day; children seen through the day at PRI would have open access to Ninewells. The driver for change was the challenge in providing services in two hospitals in Perth and Dundee 23 mile apart, and services were moved to Dundee. A 24-hour inpatient service was provided in Dundee for all children in NHS Tayside after April 2005. The analysis included data between May 2000 and April 2010.

#### Elgin (Dr Gray’s Hospital, NHS Grampian)

**Fig. 1:**
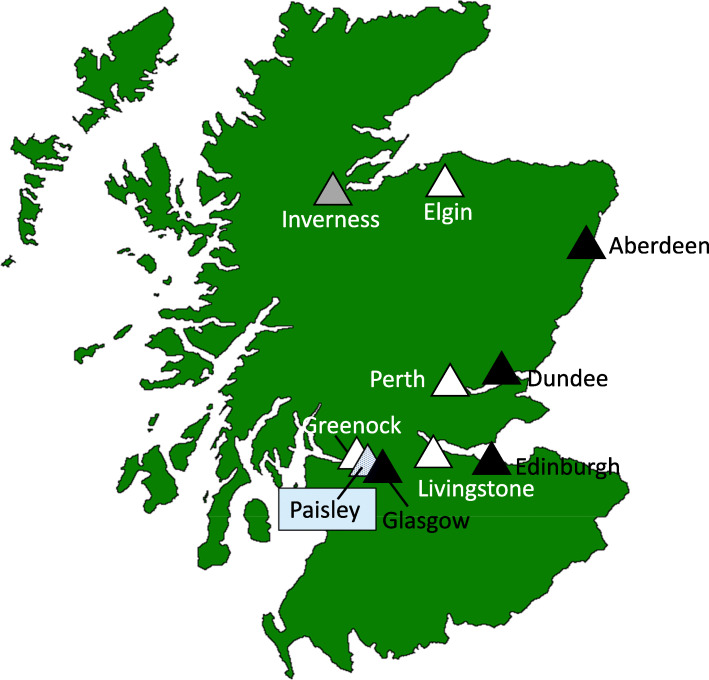
Map of Scotland identifying where the inpatient facilities (hospitals) where paediatric services were reconfigured and the adjacent inpatient facilities where services were accommodated. White triangles indicate locations where inpatient facilities were reconfigured. Black triangles indicate the location where round-the-clock inpatient services were provided post reconfiguration in an adjacent hospital. The grey speckled unit is Paisley where reconfiguration first took place in an adjacent inpatient facility (Greenock) and then in Paisley itself after which all services were moved to another facility (Royal Hospital for Children, Glasgow). The plain grey triangle identifies Inverness as a hospital geographically closer to Elgin than Aberdeen. Map outline by Topbanana (talk) - Own work, Public Domain, https://commons.wikimedia.org/w/index.php?curid=83972851

Round-the-clock inpatient services ceased in March 2018 whereafter a short stay assessment unit staffed by a combination of paediatric and emergency department physicians provided a service between 8am and 10 pm. The main driver for reconfiguration was a shortage of medical staff. Ambulatory care was provided by the hospital in Elgin where possible (e.g. parenteral antibiotics administered on an outpatient basis for febrile children). An inpatient service was provided by Aberdeen 65 miles away (the second hospital in NHS Grampian). Data were analysed between April 2013 and December 2018. The number of admissions to Raigmore Infirmary (Inverness, NHS Highland) which is only 37 miles from Elgin (Fig. 1) were also analysed between April 2013 and December 2018.

#### Paisley (Royal Alexandra Hospital, Greater Glasgow and Clyde)

All inpatient facilities ceased after reconfiguration in December 2017. The main driver for reconfiguration was the move of the largest inpatient facility in Scotland (Royal Hospital for Children, RCH) to a new site 6 miles away. No resources were put into the community in Paisley. Data between January 2013 and February 2020 were analysed. At RCH, throughout 2013–2019 many referrals are seen and discharged by paediatric emergency department staff and are not recorded as an inpatient.

#### Livingston (St John’s Hospital, NHS Lothian)

Due to an ongoing shortage of medical staffing, in July 2017 a short stay assessment unit was opened, including a facility for daily systemic antibiotic treatment. All unscheduled inpatient services had previously ceased for 3 weeks in July 2014 and for 6 weeks July and August 2015. In summer 2019 24-h inpatient services were provided 4 days a week. Data between August 2012 and March 2019 were used to analyse the impact of the reconfiguration in July 2017.

### Data analysis

The prevalence of emergency admissions/1000 children/month was calculated. Per capita standardisation was achieved using the number of individuals aged < 16 years resident in the surrounding local authorities using annualised publicly-available data [8]. At the time data were analysed no estimate population data were available for 2019 and in this instance, 2018 population data were used. Population data were not available on a monthly basis. Mixed level models were used to describe the change in admissions in the 12 months before and 12 months (or less than 12 months for more recent reconfigurations) after reconfiguration in “reconfigured” hospitals and (separately) in “adjacent” hospitals. The model included a covariate scored 1–5 corresponding to each hospital.

Interrupted time series [9] analyses were used to study change in trends in the number of emergency admissions per month over time. The period before reconfiguration was coded as 0 and post-reconfiguration coded as 1. Time period was considered as intervention centred time variable from the time of ward closure having time 0. This facilitates to estimate the rate of emergency admission at the end of ward opening period and also at the beginning of ward closure. An interaction term between time centred variable and ward closure status was introduced to find change in the rate of emergency admission after the ward closure compared to the period when ward remained open. The hypothesis was that there was a level change (intercept) in the rate of emergency admission and also change in the rate of emergency admission (slope) over the post-reconfiguration period compared to the pre-configuration period. General linear model with Poisson family and log link function was used to estimate Risk ratio (RR) and 95 % Confidence Intervals (CI) for RR. Rate Ratio (RR) was expressed in terms of relative percentage with 95% CI. A p-value less than 0.05 was considered to be statistically significant. Stata MP ver 15 was used for statistical analysis. The model investigated possible autocorrelation between data points using Cumby-Huizinga test [8] as well as graphical methods by plotting residuals against time and adjusted for it with lags of data point included as regressors wherever necessary. A scale parameter to overcome over-dispersion was applied in order to estimate appropriate Standard Error(SE) in the model. Seasonality in emergency admission was adjusted for through a Fourier term (Pairs of sine and cosine) functions [10].

## Results

### Change in admission prevalence in individual hospitals after reconfiguration

In the months after reconfiguration, the average number of admissions fell in 4 and rose in one hospital where the reconfiguration took place, Table 2. Across all the reconfigured hospitals there was a mean fall in admissions/month of 117 [95 % CI -156, -78] *p* < 0.001 and for adjacent hospitals there was a mean rise in admissions/month of 82 [95 % CI 32, 131] *p* = 0.002. For all adjacent hospitals except Aberdeen, the average number of admissions per month rose by > 7 % (i.e. twice the national annual rise in admissions across Scotland 2000–2013 [10]).

**Table 2:** Average monthly number of admissions in the year before and the year after service reconfiguration in all affected units. *Data in nine months post reconfiguration were analysed

Hospital where services were reconfigured			Hospital where services inpatient services were moved to post reconfiguration			% change in admissions/month in adjacent hospital after reconfiguration
	Monthly admissions before reconfiguration	Monthly admissions after reconfiguration		Monthly admissions before reconfiguration	Monthly admissions after reconfiguration	
Inverclyde Royal Hospital (Greenock)	108	0	Royal Alexandra Hospital (Paisley)	191	253	+32%
Perth Royal Infirmary (Perth)	66	74	Ninewells Hospital (Dundee)	446	479	+7%
Dr Gray’s Hospital (Elgin)	102	69*	Royal Aberdeen Children’s Hospital (Aberdeen)	235	238*	+1%
			Raigmore Infirmary (Inverness)	297	321*	+8%
Royal Alexandra Hospital (Paisley)	351	0	Royal Hospital for Children (Glasgow)	2358	2630	+12%
St John’s Hospital (Livingston)	539	405	Royal Hospital for Sick Children (Edinburgh)	503	571	+13%

### Change in admission prevalence across paired hospitals after reconfiguration

**Fig. 2:**
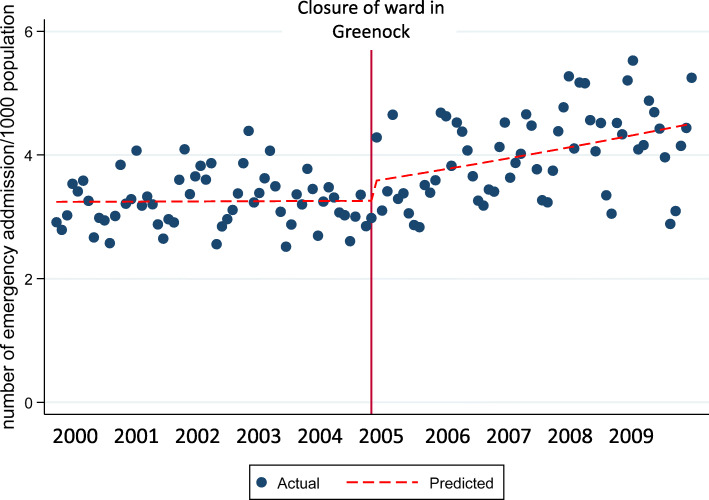
Scatter plot showing the combined number of emergency admissions per month to hospitals in Greenock (Inverclyde Royal Hospital) and Paisley (Royal Alexandra Hospital) before and after the ward in Greenock closed in November 2004. The vertical red line identifies the point in time when the closure occurred. The broken red line identifies the trend in admissions before and after the ward closure in Greenock

**Fig. 3:**
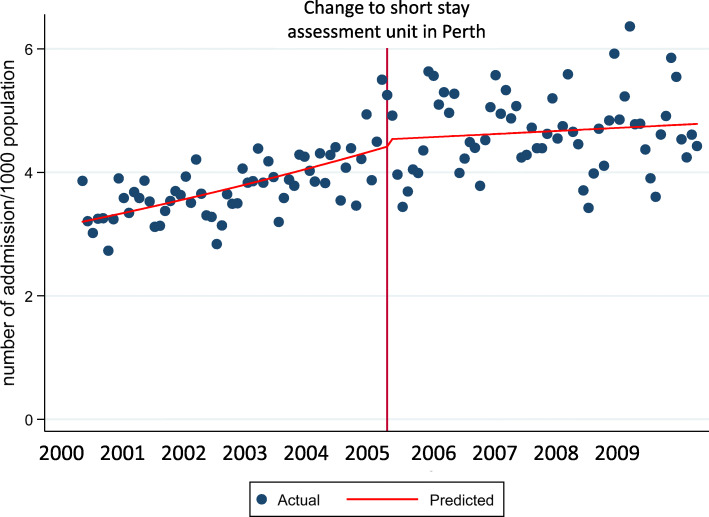
Scatter plot showing the combined number of emergency admissions per month to hospitals in Perth (Perth Royal Infirmary) and Dundee (Ninewells Hospital) before and after the ward in Perth changed from open 24 h to a short stay assessment unit open 8am-8 pm in April 2005. The vertical red line identifies the point in time when the closure occurred. The broken red line identifies the trend in admissions before and after the ward closure in Perth

There was no rise in admission prevalence to inpatient facilities in Inverclyde and Paisley prior to reconfiguration in Inverclyde but there was a rise in admission prevalence in Paisley post-reconfiguration, Table 3; Fig. 2. In contrast, there was a rise in admission prevalence across NHS Tayside (i.e. Perth and Dundee combined) before reconfiguration of services in Perth but this was followed by a fall in admissions to Dundee post reconfiguration, Table 3; Fig. 3. Before reconfiguration in Paisley there was a trend for a significant fall in admissions across Paisley and Glasgow (mean reduction 0.15 % per month [95 % CI -0.01, 0.33]) and this trend was not different post reconfiguration, Table 3. Before reconfiguration in Livingston there was a rise in admission prevalence across Livingston and its adjacent hospital in Edinburgh (mean increase 0.36 %/month [95 % CI 0.16, 0.56]) and there was no change in this rise post reconfiguration, Table 3. There was no difference in admission prevalence before or after reconfiguration in Elgin.

**Table 3:** Regression output for interrupted time series model

	Emergency admission /1000 population at the end of the period of analysis	Percentage change (95% CI) in rate of emergency admissions/month before reconfiguration	Percentage change (95% CI) in rate of emergency admission immediately after reconfiguration	Regression coefficient (95% CI) for interaction term describing the change in emergency admission/month before and after reconfiguration	*p*-value of interaction term
Admissions to Greenock and Paisley before and after reconfiguration in Greenock	3.3	0.05 (-0.01, 0.21)	6.41 (-1.6, 15.0)	0.37 (0.16, 0.60)	<0.001
Admissions to Perth and Dundee before and after reconfiguration in Perth	4.3	0.47 (0.32, 0.62)	6.99 (-.02, 14.6)	-0.05 (-0.07, -0.03)	<0.001
Admissions to Elgin and Aberdeen before and after reconfiguration in Elgin	5.9	0.12 (-0.03, 0.26)	-11.80 (-25.0, 3.7)	1.11 (-1.55, 3.84)	0.414
Admissions to Paisley and Glasgow before and after reconfiguration in Paisley	3.1	-0.15 (-0.33, 0.01)	-2.8 (-11.7, 6.9)	0.49 (-0.70, 1.66)	0.244
Admissions to Livingston and Edinburgh before and after reconfiguration in Livingston	3.4	0.36 (0.16, 0.56)	-9.77 (-21.5, 3.7)	0.41 (-0.06, 1.40)	0.418
Admissions to Inverness before and after reconfiguration in Elgin	7.5	0.12 (-0.03, 0.28)	8.1 (-2.7, 20.0)	0.21 (-0.08, 1.24)	0.681

## Discussion

The aim of this study was to determine whether trends in admission prevalence changed after reconfiguration of inpatient services. The main finding was that there were heterogeneous outcomes post-reconfiguration. For example the net prevalence of admissions rose where no contingencies were put in place for a deprived community where reconfiguration took place (Inverclyde/Paisley) but admissions fell when a number of contingencies were put in place in a relatively affluent community (Perth/Dundee). A second observation of note was that in one hospital (Elgin), admissions moved “across the border” to an adjacent health board where another hospital was closer than the “within the border” hospital where admissions were due to be accommodated. This study is the first to describe the impact of reconfiguration on paediatric admissions and although based on a small number of episodes, the findings give unique insights. Reconfiguration of services across neighbouring paediatric units is being considered in several regions across the UK and these results may be useful to clinical and management staff who are involved in delivering reconfiguration.

Our findings are consistent with another study which has described changes in admissions post reconfiguration in an adjacent inpatient facility, and where closure of one of five emergency units was associated an increase in admissions to three of the four remaining units [6].

Our study found the number of admissions to Paisley after reconfiguration of inpatient services at Greenock was greater than the sum of predicted admissions to Paisley and Greenock, but this pattern was not seen after reconfiguration in the other four hospitals, in fact admissions fell in Dundee after the reconfiguration in Perth. The fact that there were heterogeneous outcomes after reconfiguration within a single nation suggest that there are several different factors driving admissions to an inpatient facility after reconfiguration in a neighbouring hospital. For example the closure of Paisley (in the same health board as Greenock) was not followed by increased admissions so factors such as distance to “adjacent” hospital and deprivation may explain the differences after closure in the two inpatient facilities in Greater Glasgow and Clyde.

Reconfiguration is thought to have a greater negative impact on more deprived communities [6], possibly due to increased burden of illness and/or increased costs in travelling to an adjacent hospital, and there were increased admissions in Paisley after Greenock (among the 10 % most deprived communities) closed but falling admissions in Dundee after reconfiguration in Perth (among the 5 % least deprived communities).

Perhaps not surprisingly, distance from a place of reconfiguration to the nearest inpatient facility may affect where admissions are “relocated” to post reconfiguration. Although Inverness is in a different health board to Elgin and Aberdeen, after reconfiguration in Elgin there was no change in admission numbers in Aberdeen (65 miles away) but there was a rise in admissions to Inverness (37 miles away). In contrast the distances between Greenock and Paisley and Perth and Dundee are almost identical but Paisley and Perth had opposite trends in admissions post reconfiguration so where there is no alternative hospital, the distance between paired hospitals after reconfiguration in one hospital does not appear to influence admissions.

Although the number of reconfigured facilities was small there was no evidence of different admission outcomes when inpatient facilities ceased altogether compared to a change to a limited service. There was a possibility that a large difference in admission prevalence between the paired hospitals was relevant to post-reconfiguration admissions and found no evidence to support this paradigm.

A strength of our study is that it included data before and after services were reconfigured in 5 hospitals and this gives our results a wider generalisability. Our analysis adjusted for the underlying rising trend in emergency admissions to hospital [11] and also seasonality of admissions.

Our study has some limitations which should be considered. Reconfiguration in Elgin, Livingston and Paisley took place in 2017 and 2018 meaning that details of admissions after reconfiguration were limited to less than two years so it is possible that there may be a delayed change in admissions prevalence. Although there was a discrete date when inpatient services were reconfigured, it is likely that in the knowledge that services were to be reconfigured referral pathways may have changed before the official date of reconfiguration, especially where in patient services have been suspended temporarily before a more permanent reconfiguration (as was seen in Livingston). The analysis did not pool data for all hospitals in a single interrupted time series analysis because the results for different pairs of reconfigured and adjacent hospitals were heterogeneous (Table 3), but data were pooled from reconfigured and adjacent hospitals (Table 2) to reduce false positives from multiple testing but we acknowledge that the results in individual hospitals were heterogeneous (e.g. admissions rose in Perth post reconfiguration). Our analysis focussed on characteristics of the patient’s local community and healthcare system (e.g. deprivation, admission rates, distance to hospital, nature of reconfiguration) and not the individual patient. Deprivation is linked to patient characteristics such as ethnicity, and availability of and engagement with healthcare. However, we acknowledge the limitation that we are not able to determine patient characteristics such as age and diagnosis, and this could be a useful aim for future research.

## Conclusions

In summary this study used routinely acquired data to describe changes in admission prevalence after reconfiguration of inpatient services. Planners considering in patient service reconfiguration in future should be aware that admissions will change before and after reconfiguration. Contingencies which could minimise the impact of service reconfiguration on the community’s healthcare provision include having an ambulatory paediatric service (including an outpatient antibiotic therapy service), daily rapid review clinics and providing parents whose children are seen during working hours with a phone number to call if they become worried. The previously mentioned contingencies could be especially effective when a reconfigured unit is in an area of deprivation, but such contingencies could be considered as good practice for all units (reconfigured or not). Further research is required to determine whether the increased activity in adjacent hospitals post reconfiguration leads to improved patient-centred outcomes.

## Data Availability

The dataset used and analysed during the current study is available from the corresponding author on reasonable request.

## Abbreviations

CI: Confidence Interval
NHS: National Health Service
RCH: Royal Hospital for Children
RR: Relative Risk
SE: Standard Error
SIMD: Scottish Index of Multiple Deprivations
